# The Translucent Filling Cement*A paper read before the Liverpool and District Odontological Society at Liverpool on Monday, March 15, 1920. Reprinted from *Dental Record*, September, 1920.

**Published:** 1920-10

**Authors:** F. N. Doubleday

**Affiliations:** Assistant Dental Surgeon to Guy’s Hospital


### THE TRANSLUCENT FILLING CEMENT *

BY F. N. DOUBLEDAY, M.R.C.S., L.R.C.P., L.D.S.ENG., ASSISTANT DENTAL SURGEON TO GUY'S HOSPITAL

The first medical aphorism enunciated by Hippocrates is as follows: "Life is Short, Art Long, Opportunity Fleeting, Experiment Slippery, Judgment Difficult." He who attempts to investigate translucent cement fillings learns by experience its truth. Several times I have been about to write to your council asking permission to change the subject of this evening's meeting. That has not been done, because—whatever may be the deficiencies of this paper—it appears to me that a discussion of the subject is due, and however little is here offered to that end your experience will make the evening a profitable one.

* A paper read before the Liverpool and District Odontological Society at Liverpool on Monday, March 15, 1920. Reprinted from *Dental Record*, September, 1920.

My aim has been to prepare a consideration of the factors upon which the manipulation of these fillings depend and to inquire into their possible effects upon the live pulp. The first part of this paper contains a recapitulation of the investigations which have been carried out by other workers upon the chemical composition of this class of filling material; the second part is an account of some original experiments which have been carried out to endeavor to elucidate the problem of their action upon the living pulp and the conclusions which were the outcome of this work. For this purpose I chose four different makes of translucent filling and to these added an oxyphosphate of zinc cement—one which is in very general use—for the purpose of comparison. These are numbered from 1-5. No. 1 is a well known filling of Swiss origin; Nos. 2 and 3 are made from American formulæ, and No. 4 is of English manufacture. The problems which confronted me were these: what are the end products formed when the cement is fixed by the dentist; is there any chemical or physical factor in the end composition; which is likely to have an adverse effect upon a live tooth pulp?

The first question is one which can only be properly solved by quantitative analysis by an expert chemist. Here I was fortunate in having access to some expert analyses which had been independently done and these confirmed the general results already published, and it appears that they form a safe guide as to the approximate composition and character of the powder and liquids. I have not found anything which would lead me to believe that there is any essential difference between the various makes of filling materials. I have inquired from a good many expert dentists as to their views of the possibility of translucent fillings acting adversely upon the pulp; some extremely able men are convinced that they have no experience of adverse effects; others, who are equally competent to give their opinion, are certain that pulps do die under these fillings if the cavities are not lined by some indifferent material.

In considering filling materials, apart from metallic fillings such as gold and amalgam, there remains at the one extreme the oxyphosphate cements, and at the other the porcelain fillings obtained by fusion of aluminium and silicates, in an electric furnace. The synthetic fillings represent a compromise between these extremes; an attempt to combine the tooth-like lustre of the aluminium and silica with the ease of manipulation and adhesiveness of a cement. The translucent fillings are those which are made by combining aluminium or other acid phosphates in solution with various bases. It is interesting to note that the earliest form of these cements was suggested and patented by that well-known English dentist, Fletcher of Warrington, in 1879. His preparation consisted of calcium, aluminium and silica fused together, then powdered and mixed with acid aluminium phosphate. Later preparations follow this line but with considerable modifications. The patent specifications of a German translucent cement are as follows: "The precipitate obtained by adding sodium silicate to a solution of basic beryllium nitrate is allowed to remain in water a certain length of time and is then filtered, washed carefully, dried and heated to low redness. The preparation thus obtained (the empirical formula of which is BeO. $SiO_2$) is very finely ground and is then used as thus prepared, or if greater hardness is desired, it has intimately mixed with it some powdered glass or alumina. This powder is carefully mixed with fifty-two per cent. phosphoric acid in which aluminium phosphate is dissolved nearly to the point of saturation and to which liquid a slight amount of zinc or strontium phosphate is also added. The reaction occurring during hardening may be interpreted as follows: the acid liquid withdraws from the basic beryllium silicate its beryllium oxide content, with the precipitation of phosphates and the hydration of the silicate.

The following abstracts of some English patents are worth consideration.

1905. Patent No. 11,181. Basic or neutral phosphates, pyrophosphates and borate of aluminium is mixed with solution of orthmeta and pyrophosphoric acid to form a paste. Aluminium hydrate may be mixed with the phosphate. Aluminium hydrate, zinc oxide or magnesium oxide or mixtures of these may be dissolved in the phosphoric acid before the latter is mixed with the phosphate or borate.

1906. Patent No. 2625 (Rawitzer's Astral). The mixture is unfused and contains aluminium silicate and calcium, alum and silicon oxide. For use the material is mixed with solution of phosphoric acid containing alumina.

1907. Patent No. 20304 (Schoenbeck). Cryolite, silicate acid and calcium oxide are fused with five per cent. beryl. The cooled mass is powdered, and for use is mixed with phosphoric acid containing a little aluminium hydoxide dissolved in it.

The following analyses, made by expert chemists, have been published.*

The powder contained:

| | |
|---|---|
| Silicon dioxide ($SiO_2$) .. .. .. .. | 36.91 |
| Aluminium oxide ($Al_2O_3$) .. .. .. .. | 33.69 |
| Phosphorous pentoxide ($P_2O_5$) .. .. .. | 4.00 |
| Calcium oxide (CaO) .. .. .. .. | 7.26 |
| Sodium oxide ($Na_2O$) .. .. .. .. | 15.88 |
| Water (hydroscopic) .. .. .. .. .. | 2.26 |
| | 100.00 |

* C. C. Voelker—"The Place of the Silicates in Dentistry."—*Dental Summary,* 1916, p. 177.

The liquid proved to be a modified phosphoric acid solution, containing zinc aluminium phosphates.

Another contained:

| | |
|---|---|
| Calcium oxide | 2 parts |
| Silicon dioxide | 44 ,, |
| Aluminium oxide | 41 ,, |
| Sodium oxide | 13 ,, |
| | 100 parts |

The liquid was a solution of phosphoric acid, containing about ten per cent. hydrofluo-silicic acid.

A German analysis of Harvardid published in 1913 gives the following:

| | |
|---|---|
| Silica ($SiO_2$) | 40.26 |
| Alumina ($Al_2O_3$) | 50.62 |
| Lime (CaO) | 5.00 |
| Magnesia (MgO) | .51 |
| Potassium and sodium oxide | 1.08 |
| | 97.47 |

From these analyses it appears that the translucent fillings are mainly compounds of aluminium and calcium, with phosphoric acid, often modified by acid phosphates in solution and by water. It is helpful to consider these elements as they are found in a crude state in nature.

Aluminum has an atomic weight equal to 27.81. Its oxide $Al_2O_3$ forms corundum with which we are so familiar, and also occurs in rubies and sapphires. Turquoise is an hydrated phosphate of aluminium; the clays are mainly silicates and the feldspars are compound silicates of aluminium. The metal is also found in combination with beryllium, as chrysoberyl, beryllium alumate $Al_2O_3$, BeO. Beryl is a double silicate of aluminium and beryllium having the formula 3 BeO, $Al_2O_3$, 6 $SiO_2$.

Silicon, Si., has an atomic weight equal to 28.4. As the oxide $SiO_2$ it occurs in flint, sand and quartz. Carborundum is a compound of carbon and silicon. In the preparations which we are considering it may occur in the form of the oxide $SiO_2$ or as a part of the acid radicle.

Silicon dioxide will form weak acids. Orthosilicic acid has the formula $SiO_2$, 2 $H_2O$. The acid is a colloidal substance. Calcium appears to be used in the form of an oxide, which by combining with the phosphoric acid in the liquid aids the setting of the cement.

Beryllium is a metal with an atomic weight equal to 9.1. Emerald is a form of beryllium which also occurs in nature as a beryllium silicate $Be_2$ $SiO_4$ and as a double silicate 3 BeO, $Al_2O_3$, 6 $SiO_2$, the soluble beryllium compounds are characterized by possessing a sweetish taste.

The liquid of the earlier cements was ortho-phosphoric acid, $H_3PO_4$. Phosphoric acid is tribasic, forming three series of salts in this manner:

| | |
|---|---|
| Di Hydrogen sodium phosphate .. .. | $H_2Na\,PO_4$ |
| Hydrogen di sodium phosphate .. .. | $H\,Na_2PO_4$ |
| Tri sodium phosphate .. .. .. .. | $Na_3PO_4$ |
| (Normal sodium phosphate.) | |

Of these the second is neutral, and the third definitely alkaline, in its reaction. Similar series of salts are formed with aluminium or calcium bases, but their formulæ are more complex. The orthophosphoric acid in modern cement liquids is greatly modified by the addition of acid phosphates of aluminium and certain other metals. Probably the bulk of them contain a considerable portion of water, acid phosphates in solution, and free phosphoric acid which is included to initiate the chemical interaction of the powder and the liquid and to hasten the period of setting.

The next point to which our attention must be directed is the nature of the reaction between powder and the liquid; what is its character? What are its end products?

This is a complex problem, but we are greatly aided by some published observations of Dr. T. Martin Lowry, R.F.S., Professor of Chemistry in the University of London and the Director of our Chemical Department at Guy's Hospital.

Professor Lowry puts it thus: "The phenomena of setting have been studied somewhat fully in the two leading cases of plaster of paris and of Portland cement. In the former case it has been shown that the setting involves a two-fold crystallization, the half hydrate, 2 $CaSo_4$, $H_2O$ giving first a metastable and then a stable form of the di-hydrate, $CaSo_4$, 2·$H_2O$. In the case of Portland cement the decomposition by water of calcium silicates and aluminates containing an excessive proportion of lime is probably the fundamental change, but the actual mechanism of the setting and the extent to which colloidal material plays a part in it appear to be still matters of controversy.

"In general, the properties demanded of dental cements are similar to those which are essential in Portland cements used for constructional work, the two most important properties being (1) hardness, which in this case must take the form of 'edge strength,' and (2) smallness of change of volume in setting, which in the case of a dental cement would produce a loose filling if there were a substantial construction, and a split tooth if there were a substantial expansion. A principal point of difference is found in the fact that a higher rate of setting is required in a dental cement, since, whilst the boarding of a concrete structure may conveniently be kept in position for a day or more, these conditions can scarcely be applied to the insertion of a stopping in a tooth."

Professor Lowry classifies cements in this way.

"(*a*) Zinc oxyphosphate cements in which zinc oxide is converted into basic phosphate by a solution of phosphoric acid which may already contain some zinc phosphate.

(*b*) Silicate cements, in which various silicates and various salts of aluminium are converted into phosphates by the action of an acqueous solution of phosphoric acid, sometimes saturated with aluminium or zinc phosphate.

(*c*) A caking, practically equivalent to the formation of a cement, accompanies the conversion of calcium phosphate by the action of sulphuric acid into a mixture of sulphate and superphosphates.''

During my four years as editor of the *British Dental Journal* I was carefully watching the English and foreign literature for some good information upon this subject. A paper by Dr. Vogt, of the Mellon Institute at Pittsburg, was eventually selected and published.* In the last six months the opportunity for studying and to some extent checking the published work upon translucent cements has only confirmed my opinion that this is by far the most accurate and scientific account which has yet been given of the subject we are considering. Those of you who file your journals will find it well worth re-reading. I believe that the paper represents the work done in obtaining the formula for Smith's ''Certified Enamel.''

Dr. Vogt states that after experiment he eliminated the various bases until calcium and aluminium remained as the most suitable. Of these two, calcium oxide (quicklime) is too caustic, and, in addition, is as opaque as zinc oxide, while aluminium oxide is too inactive to react with the liquid, and is also opaque. Aluminium oxide has desirable properties which would recommend it if only its unpleasant characteristics could be eliminated. Therefore, some combination of these two, or of these two along with other chemicals, must be sought.

Dr. Vogt's conclusions are as follows: ''Various modifications have been adopted so that the constituents of the present day silica may include the oxides of calcium, aluminium, beryllium, strontium, sodium, boron, phos-

---

* *British Dental Journal,* 1918, p. 693.

phorus and titanium, along with fluorides and fluosilicates, furnish the acid characteristics. However, the essential plan of a strongly basic aluminium silicate as foundation remains the same. The chief object of the modifications has been to lower the melting point of the fusion mixture enough to increase the reactivity of aluminium oxide, which bears the same fundamental relation to the silicates that zinc oxide does to the oxyphosphates. In fact, the term 'aluminophosphates' would be as descriptive as 'silicates' for this class of filling materials.''

You will observe that this agrees with Professor Lowry's conclusions upon the end products.

The reaction between the powder and the liquid are probably as follows: The phosphoric acid reacts with the powder producing acid phosphates of aluminium and any other bases, such as calcium, which may be present. These acid phosphates react with the powder forming normal phosphates of aluminium and other bases and water, some of the water freed by the decomposition of the phosphoric acid reacts with the reduced silicon oxide and hydrates it.

Let us now consider the possibility of translucent fillings acting adversely upon the pulp. In this connection the first consideration seemed to lie in the possible presence of arsenic in the powder or in the liquid. Arsenic is a common impurity both of the bases and of the acid as they are originally obtained by the chemical manufacturers; they, however, take special measures to remove it. When the dental cement makers deal with the several ingredients they also carefully test for and remove any traces of arsenic. If you consider how greatly it is to their interest to do so, it appears to me that the chance of arsenic being present when the cement reaches us is very small. In the specimens which I have tested myself and had tested by others, the presence of arsenic in a soluble form has not been detected. The next danger appears to be in the possible presence of acid reacting substance in the mixed cement, when and after it is inserted into the tooth cavity. Here I believe that we have a very real danger. Acid states of the body favor catalytic or breaking down processes. In diabetes and in dental caries we have examples of general and local diseases in which acid states are the constant pathological factor.

The preliminary experiments which I carried out seemed to show that the remnants of filling materials mixed for actual use in practice frequently showed an acid reaction to litmus paper which continued for some time. The experiments which I am now going to show you were devised to confirm this observation and were chosen as giving standard reactions capable of being easily checked by any dentists who care to confirm or refute the accuracy of the observations. I have here a series of test-tubes each containing 5 c.c. of distilled water which is neutral in its reaction to litmus. To each has been added three drops of decinormal litmus solution. Here are a series of five numbered cards upon which the mixed filling materials from each variety will be placed, with a note of the time when the mix is made. In each case I shall endeavor to make a thorough mix according to the directions of the several manufacturers. Each mix is divided into three portions; portion A is immediately dropped into a test-tube of litmus and you will see that in each case it turns immediately pink. Portion B is rolled into a ball, covered with vaseline, at the end of ten minutes the vaseline is removed and the pellet dropped into another test-tube of litmus, you will again observe that in each case it turns pink at once, showing a well-marked acid reaction. Portion C is packed into a neutral glass tube 5 mm. in length, its ends are sealed with vaseline and it is set aside for one hour; at the end of this time No. 5, the oxyphosphate of zinc, does not affect the blue litmus; No. 1 has a slowly acid reaction; Nos. 2, 3 and 4 all change the color of the litmus immediately. It is of course an easy matter working here under the eye of so many people to introduce fallacies into the tests, but, as was observed before, the experiments were purposely planned to make checking easy; if you will be kind enough to repeat the observations yourselves at the end of the evening, or to do them independently at home, I think you will confirm my results. What do they imply? I had not previously realized—and the textbooks do not state the point—that oxyphosphate of zinc cements are acid when inserted into a tooth cavity. In the case of zinc the interaction of the powder and the liquid takes place fairly quickly, so that at the end of a short time the acid reaction goes. The zinc powders react rapidly and completely with the acid liquids. Aluminium on the contrary is chemically inert, and the reaction of its compounds with the liquids is slow, so that the mixed filling remains acid for a long time. I have here a piece of blue litmus paper upon which I will place four separate drops from the four translucent cement liquids, each drop coming from a separate pipette of the same size and make. I think you will agree that No. 4 causes a much deeper red stain than the others. No. 2 appears to come next, while judged by this rough test—which is, however, easy for you to verify—Nos. 1 and 3 are made from liquids which are the least acid at the commencement of the reaction. These reactions seem to show that the pulp must be more tolerant to acid than I had believed to be the case, but were the acid reaction continued over a long time, as it appears to me must frequently occur with many translucent fillings of all makes, that the acid reaction is very likely to lead to death of the pulp if the cavity is deep and the filling near to that tissue.

Another possibility of danger to the pulp seems to lie in the transmission of thermal change to the pulp; either by leakage at the margin of the filling or by the conductivity of the mass of the set filling. I have here some glass tubes 2.5 cms. in length; these have been filled with carefully prepared mixes of the several fillings and then their ends were sealed with vaseline; at the end of ten minutes the vaseline was removed, and they have been immersed in bottles of Swan fountain pen ink, from which I will now remove them. You will notice first that shrinkage appears to be practically absent, if one attempts to press the plug of the filling out of the tube it comes away easily in each case; but the lack of adhesion appears to be inherent in the filling and not due to its shrinkage from the walls of the tube. A second series of shorter tubes contain a series of the four translucent fillings hurriedly and imperfectly mixed and then treated as before. Here too there is very little penetration by ink. These imperfectly mixed fillings differ in two important respects from those which were more perfectly made; firstly, their acid reaction is more marked when the mixing is completed and lasts for a much longer time; secondly, I will ask you to observe the difference in the appearances of the mix as shown in the thin films under the microscope. Particles of the filling materials, both good mixes and bad, have been upon microscope slides, a cover slip was pressed on to them as firmly as possible, and they were then ringed with a neutral varnish, labelled and allowed to set. Comparing the bad mixes with the good ones, it is at once noticeable that a large number of bubbles of liquid remain in the poor mixes; in the good ones liquid bubbles are very few. This seems to show that in a poor mix a relatively large quantity of uncombined liquid will remain in the set filling, thus accounting for its increased acidity and also rendering it much more liable to disintegration by the fluids of the mouth; the probability of the latter occurrence is obviously largely increased where the filling is allowed to become moist within the ten minutes necessary for the primary setting. My conclusions upon this heading are that none of the fillings shrink in such a manner as to allow primary leakage of fluids at their margin, but that they probably soon do so as the result of wastage, where they have been imperfectly mixed or not allowed to undergo primary setting under proper conditions.

Secondly, there is the problem of the conductivity to thermal change of the set fillings. For this purpose I devised the following experiment. A good mix of filling was made and packed into a No. 2 celluloid jiffy tube into which while still soft was passed the platinum portion of an unconnected D. M. Co. cautery point; the ends of the tube were sealed with vaseline and the filling allowed to set. At intervals, varying in different cases from ten minutes to several days, the celluloid cap was removed, pellets of wax placed along the filling; then the cautery was heated and the time which elaspsed before the wax melted was noted. As a means of comparison the experiment was not a success, the wax, even on the oxyphosphate of zinc fillings, melted much more quickly than I expected, and showed no appreciable difference in thermal conductivity between that and the translucent fillings or between the several members of the translucent fillings. If anyone here would follow up these experiments, using a graduated resistance to heat the cautery and wax of different melting points upon the fillings, I think that useful results would be obtained.

A further minor point of interest is the question of crystallization. Some of the manufacturers make a great point of the presence of crystals in their fillings. These I have not been able to detect as they are described. Some slides have been prepared in which the filling material was mixed with definitely crystalline substances, in one case sifted sugar and in the other salt. Comparing these films with normal ones shown under the other microscopes it seems that the films are largely amorphous, and examination under a polarizing microscope confirms the observation. In the films mixed with sugar and salt the crystals of these substances may be most definitely seen, but the formation of large crystals breaking up into smaller ones is a matter which I have not been able to follow. One would expect a definitely crystalline filling to be unsuitable for its purpose, the constant refraction of the light from the surfaces of the individual crystals would result in their being either lost by refraction in the filling or reflected from its surface. A diamond would not make a satisfactory translucent filling; the physical properties of an amorphous substance like glass is what seems more likely to meet the physical requirements for the transmission of light.

Another point which is worthy of note is that of the discoloration of translucent fillings. Aluminium salts are mordant dyes, and as such are used for fixing stains in the process of calico printing. It seems probable that acting in this way they may fix organic stains which come in contact with them, such, for example, as the red coloring matter in carbolic tooth pastes.

The conclusions at which I have arrived are as follows: (1) That all cement fillings exhibit a marked acid reaction and probably lead to the slow death of the pulp in deep-seated cavities. (2) That this acid reaction is more marked and lasts much longer in the alumino-silicate translucent cement fillings. (3) That translucent fillings rapidly disintegrate unless the manufacturers' instructions regarding the care of the original constituents, their mixing, and the avoidance of moisture during the ten minutes of preliminary setting are carefully observed.

